# Attitudes of primary care physicians towards antimicrobial stewardship and the impact of a multi-part training course – a pilot study

**DOI:** 10.3205/dgkh000450

**Published:** 2023-10-10

**Authors:** Katharina Last, Arne Simon, Barbara C. Gärtner, Sören L. Becker, Cihan Papan

**Affiliations:** 1 Institute for Hygiene and Public Health, University Hospital Bonn, Bonn, Germany; 2 Center for Infectious Diseases, Institute of Medical Microbiology and Hygiene, Saarland University, Homburg, Germany; 3 Pediatric Oncology, Saarland University Medical Center, Homburg, Germany

**Keywords:** antimicrobial stewardship, antibiotic stewardship, antimicrobial resistance, primary care, antibiotic prescribing, post-graduate medical training

## Abstract

**Background:**

A plethora of antimicrobial stewardship (AMS) programs has been initiated during the past years, focusing on hospital settings. Primary-care physicians have seldom been addressed, although the majority of antibiotic prescriptions are issued for outpatients. We sought to investigate attitudes of primary-care physicians and the impact of a customized training course.

**Methods:**

Primary-care physicians in southwest Germany were invited to a multi-part training course on AMS in the primary-care setting. Participants were asked to answer a questionnaire about their attitude and factors that hinder them from implementing AMS or enable them to perform AMS. In addition, a knowledge assessment exam at the beginning and end of the training was conducted on selected infectious diseases/syndromes.

**Results:**

In total, 36 primary-care physicians participated in the training course. The predominant age group was 51–60 years old (36%; 13/36). The majority, 23/35 (66%), indicated never having had AMS training, while 22/35 (63%) acknowledged partly implementing AMS activities in their daily routine. The primary barrier was lack of expertise, while the main motives were reducing antimicrobial resistance and optimizing patient care. The provision of guidelines was regarded as more important than feedback on their prescription behavior. Exam performance improved from the initial to the final exam on all topics.

**Conclusion:**

Customized AMS training courses are a feasible and potentially complimentary tool to address antibiotic misuse in the primary-care setting.

## Zusammenfassung

**Hintergrund:**

In den letzten Jahren wurde eine Vielzahl von „Antibiotic Stewardship“ (ABS) Kampagnen in Krankenhäusern ins Leben gerufen. Ärzte in der Primärversorgung wurden nur selten adressiert, obwohl die Mehrzahl der Antibiotikaverordnungen bei ambulanten Patienten veranlasst wird. Wir hatten das Ziel, die Einstellung von Ärzten in der Primärversorgung zu ABS und die Auswirkungen eines maßgeschneiderten Fortbildungskurses zu untersuchen.

**Methoden:**

Ärzte in der Primärversorgung im Südwesten Deutschlands wurden zu einem mehrteiligen Fortbildungskurs über ABS in der Primärversorgung eingeladen. Die Teilnehmer wurden gebeten, einen Fragebogen zu ihrer Einstellung und zu Faktoren zu beantworten, die sie an der Durchführung von ABS hindern oder sie dazu befähigen. Darüber hinaus wurde bei der Teilnahme und beim Verlassen des Kurses ein Wissenstest zu ausgewählten Infektionssyndromen durchgeführt.

**Ergebnisse:**

Insgesamt nahmen 36 Ärzte an dem Kurs teil. Die vorherrschende Altersgruppe war die der 51- bis 60-Jährigen mit 36% (13/36). Die Mehrheit, 23/35 (66%), gab an, noch nie an einer ABS-Fortbildung teilgenommen zu haben, während 22/35 (63%) angaben, ABS-Aktivitäten teilweise in ihre tägliche Routine integriert zu haben. Das Haupthindernis war der Mangel an Fachwissen, während die Hauptmotive die Reduzierung der Antibiotikaresistenz und die Optimierung der Patientenversorgung waren. Die Bereitstellung von Leitlinien wurde als wichtiger erachtet als eine Rückmeldung über das eigene Verordnungsverhalten. Die Prüfungsleistungen im Wissenstest stiegen während des Kurses in allen Themenbereichen.

**Schlussfolgerung:**

Maßgeschneiderte ABS-Fortbildungen sind ein praktikables und potenziell ergänzendes Instrument zur Bekämpfung des übermäßigen Einsatzes von Antibiotika in der Primärversorgung.

## Introduction

Antimicrobial resistance (AMR) has become the greatest infectious-disease threat to human health, with an estimated 1.27 million directly attributable deaths per year worldwide [1]. As antibiotic overuse in humans is one of the main drivers, antimicrobial stewardship (AMS; or antibiotic stewardship, ABS) programs have been widely designed and implemented in many countries. However, most of the AMS initiatives are focused on inpatients and hospital settings [2], [3], [4], while most antibiotics are dispensed in primary-care settings [5], [6], [7] [8]. Therefore, a customized approach to address AMR and antibiotic misuse in outpatients may be necessary, considering the particular demands and characteristics of primary care, i.e., time constraints, less availability and longer turnaround time of diagnostic tests, high prevalence of diagnostic uncertainty, and limited patient follow-up.

The fact that the majority of respiratory tract infections is caused by viral pathogens offers an enormous potential for curbing antibiotic overuse [9]. Urinary tract infections are another major entity which can lead to inappropriate therapy. Both entities are highly prevalent in the primary-care setting [7]. 

In Germany, a very successful and well-received continuing-education program has been offered in recent years which aimed at physicians in hospital settings (“Antibiotic Stewardship Experts”) [10]. These experts receive four modules of specific AMS training and implement an AMS intervention as part of earning an “Antibiotic Stewardship Expert” certificate. Nevertheless, there is still a dearth of large-scale interventions or educational formats to meet the particular AMS needs of primary-care physicians in Germany, with only a few exceptions [11].

We therefore aimed to pilot a project focusing on primary-care physicians in the federal state of Saarland, which leads all German states with regard to the amount of antibiotics dispensed per inhabitant in the outpatient setting [12].

## Methods

We conceptualized and conducted a pilot three-day course on AMS in the primary-care setting, aiming at general practitioners and physicians with other specialties working in primary care. The course took place over a period of four weeks in January/February 2020, with the first two half-day sessions being one week apart, and the third session following three weeks after the second. We chose Wednesday afternoons, as most primary-care physicians close their practices on Wednesday afternoon. We advertised the course in the medical journal of the federal state of Saarland (“Saarländisches Ärzteblatt”), on the website of InfectioSaar (a regional AMR network to provide courses, guidelines, and information leaflets to healthcare practitioners across different healthcare sectors; https://infectio-saar.de/), and via mailing lists. The primary aim was to include primary-care physicians from Saarland; however, physicians from neighboring regions (e.g., Rhineland-Palatinate) were also allowed to participate.

The course consisted of nine educational modules, each running for 30 to 60 minutes, covering the following topics: basics and concept of antimicrobial stewardship; antibiotics in primary care; pharyngitis/tonsillitis; urinary tract infections; respiratory tract infections; pathogen diagnostics and local resistance patterns; tools and prescribing strategies for daily use; skin and soft tissue infections; antibiotic use in children (for the course leaflet [page 1; only available in German] see Attachment 1). The modules included short lectures and case-vignette-based discussions. The second and third session began with a short repetition of the previous session.

At the beginning of the course, we asked the participants to answer a questionnaire on previous AMS experience, attitude, barriers and facilitators for their doing AMS in daily work. The questions comprised multiple-choice as well as open-text items see Attachment 1 (page 2–3; only available in German).

In addition, we conducted a formative knowledge assessment based on a short written exam with six multiple-choice questions covering the use of antibiotics and diagnostics in common situations in primary care (Attachment 1, pages 4–5; only available in German] ). The exam was repeated upon com-pletion of the course to allow a pre-post comparison.

The questionnaire and exam items were reviewed beforehand by multiple expert-level specialists on infectious diseases and AMS (KL, AS, SLB, CP).

Participation in the course and the questionnaire was voluntary, and all data obtained were anonymized. This study was exempt from institutional review board approval.

## Results

**Figure 1:**
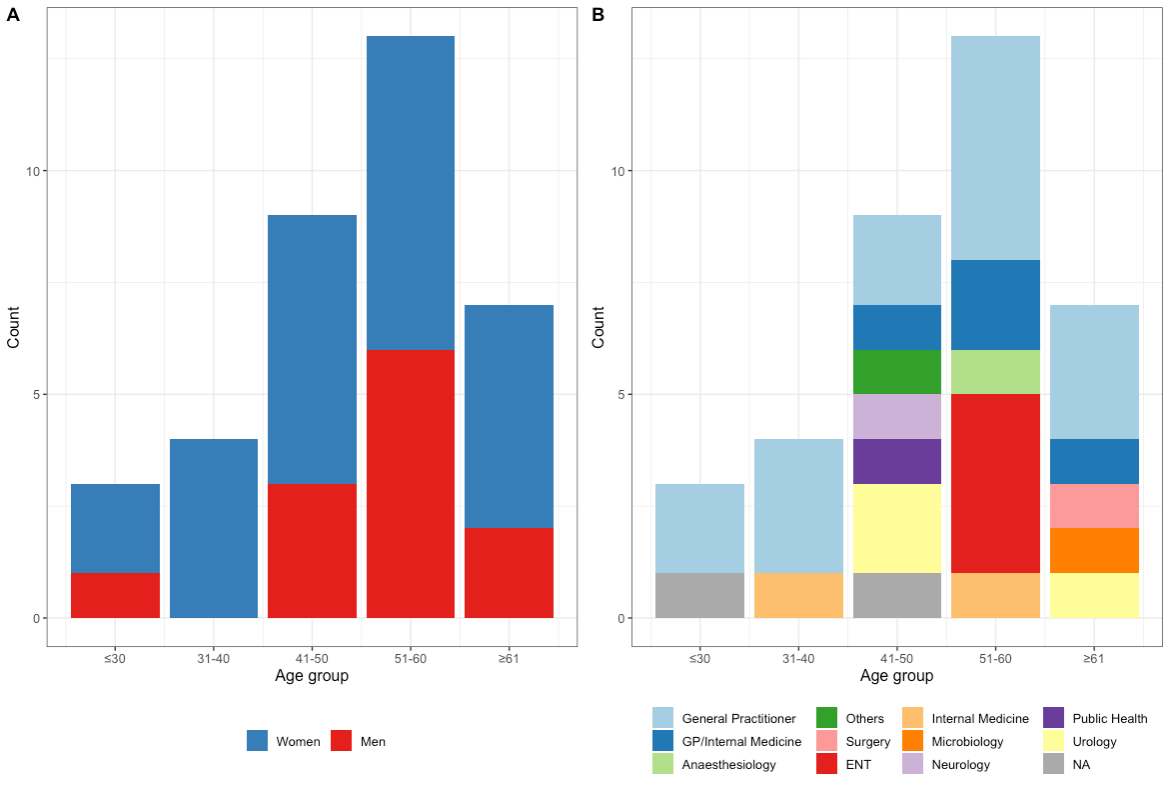
Age distribution of participants, (A) by gender; (B) by specialty; GP: general practitioner; ENT: ear, nose, throat physician; NA: not available.

In total, 36 physicians participated in the AMS for primary care course, of whom 24 were women (67%). The majority of participants was between 51 and 60 years of age (Figure 1A (Fig. 1)). Participants had a widely differing specialty background, with the predominant specialty group being general practitioners (Figure 1B (Fig. 1)).

**Figure 2:**
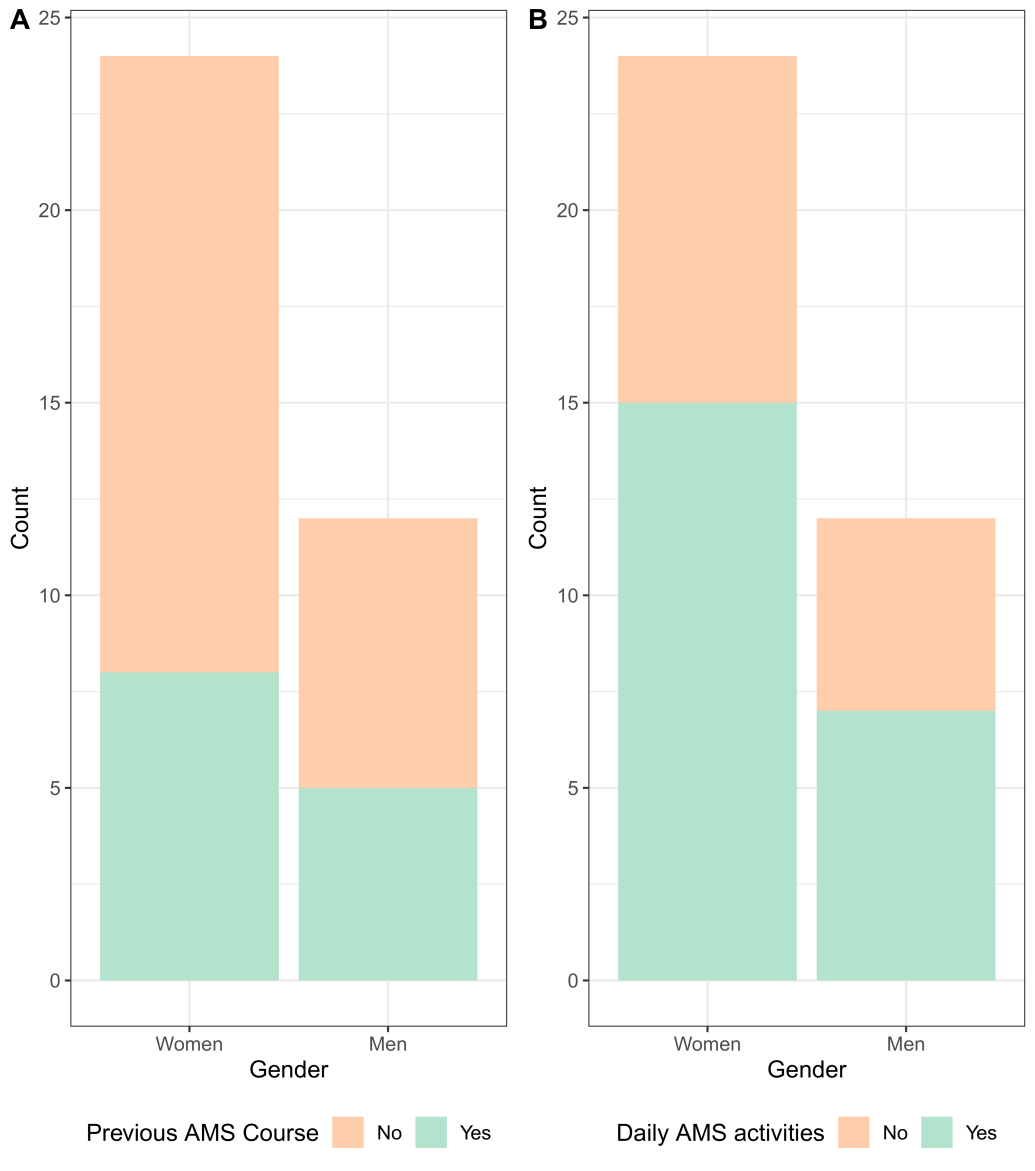
(A) Previous participation in an antimicrobial stewardship course, by gender; (B) Implementation of antimicrobial stewardship activities in daily routine, by gender. “No” included “I don’t know” (n=5 among women, n=2 among men) and NA (not available, n=1 among men).

With regard to previous experience, 13/36 (36%) indicated having attended a course on AMS in the past, with this percentage being comparable between men (5/12, 42%) and women (8/24, 33%) (Figure 2A (Fig. 2)). The proportion of participants indicating that they already implemented AMS in their daily work were comparable between the genders (women 15/24, 63% vs. men 7/12, 58%) (Figure 2B (Fig. 2)). Open text responses are shown in Table 1 (Tab. 1). 

In cases of a negative answer, we asked for reasons. Participants stated that they had “lack of expertise” in six instances; “lack of tools” was mentioned twice, while “lack of time”, “patients’ demands”, and “I don’t need it” were mentioned once each.

**Figure 3:**
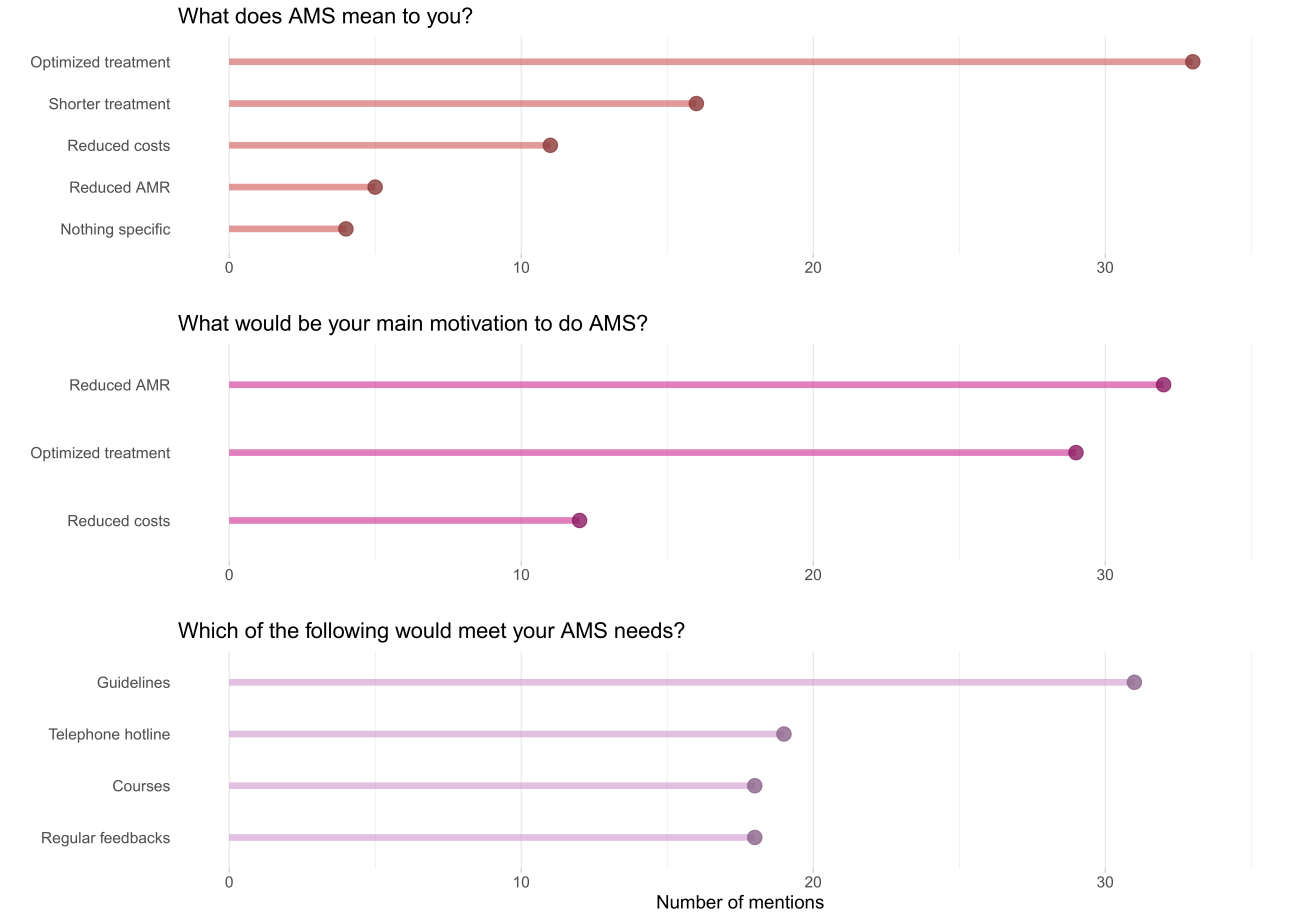
Type and number of responses to the questions “What does AMS mean to you?”, “What would be your main motivation to do AMS?”, and “Which of the following would meet your AMS needs?”; multiple answers were possible; AMR: antimicrobial resistance; AMS: antimicrobial stewardship

When asked about how they would define AMS (multiple answers possible), participants mostly chose the option “optimized treatment” (n=33), followed by “shorter treat-ment” (n=16) and “reduced costs” (n=11). Furthermore, “reduced AMR” was mentioned in the open-text box (n=5) (Figure 3 (Fig. 3)).

In contrast, “reduced AMR” was mentioned 32 times when asked about what the main motivation of the participants would be to do AMS (multiple answers possible), followed by “optimized treatment” (n=29), and “reduced costs” (n=12) (Figure 3 (Fig. 3)).

We asked the participants to choose which would best fit their AMS needs (multiple answers possible). By far, “guidelines” was mentioned the most (n=31), followed by an even distribution for “telephone hotline” (n=19), “cour-ses” (n=18), and “regular feedback” (n=18) (Figure 3 (Fig. 3)). 

This was in line with open text responses, which were often about guidelines and the role of diagnostic tests to guide the administering or withholding antibiotics, e.g., point-of-care C-reactive protein tests (Table 1 (Tab. 1)).

On the initial exam (results available for n=33), participants performed particularly well for the items Q3 (Guideline-based treatment of uncomplicated urinary tract infection), Q1 (Guideline-based treatment of community-acquired pneumonia), and Q5 (justification to treat non-specific long-term symptoms with fluoroquinolones). Percentages of correct answers for these items were 79%, 73%, and 70%, respectively. In contrast, items Q6 (probability of treating non-specific long-term symptoms with fluoroquinolones), Q2 (diagnostic and therapeutic management decision in pharyngitis with low probability of Group-A streptococci), and Q4 (localized skin and soft tissue infection) yielded a lower percentage of correct answers (27%, 15%, and 12%, respectively).

**Figure 4:**
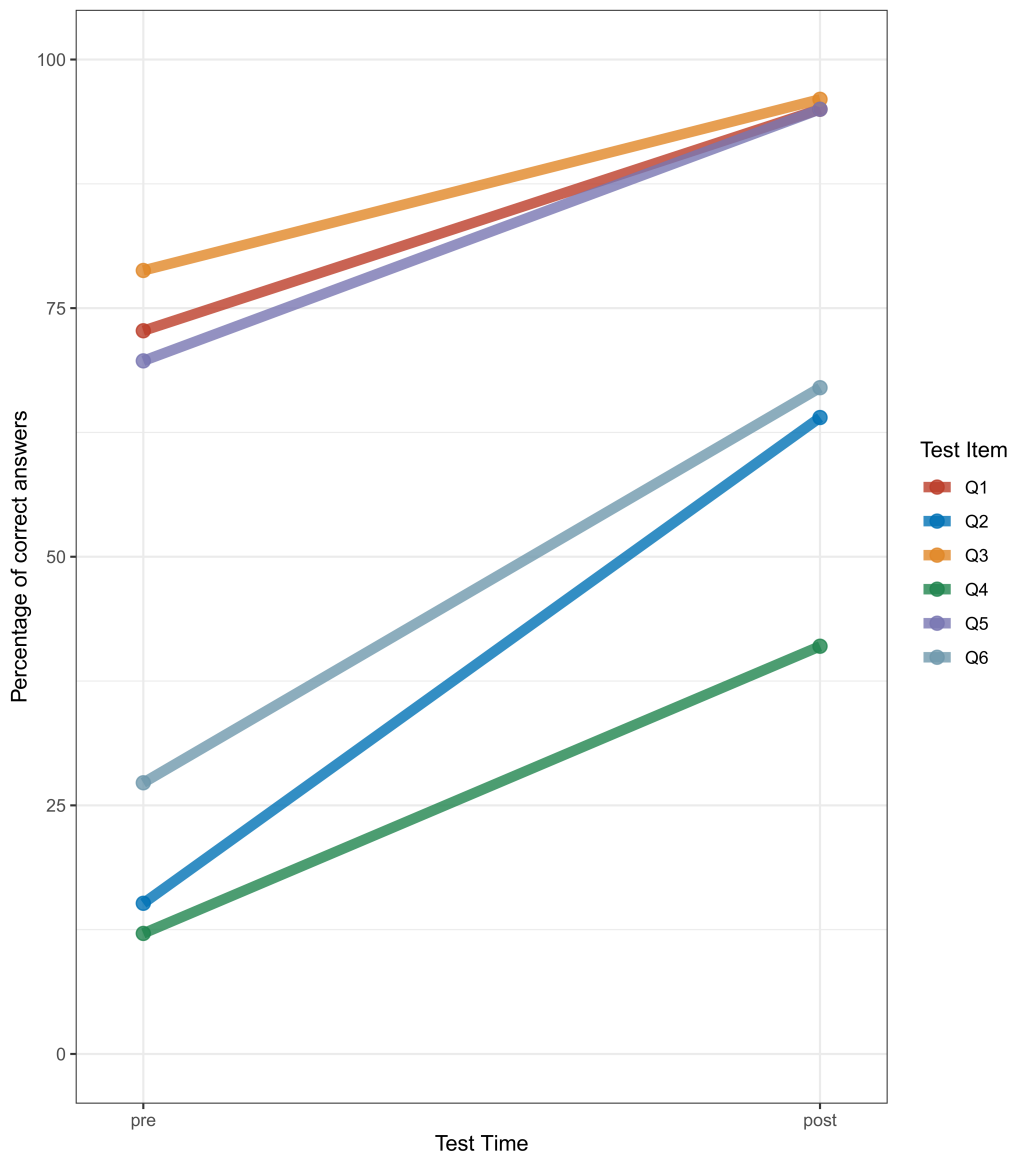
Percentage of correct answers by participants per test item and test time, “pre” indicating at the start of the course (n=33) and “post” indicating after completing the course (number of respondents per test item 22, 22, 25, 22, 24, and 24, respectively)

On the post-course exam, test performance improved across all items (Figure 4 (Fig. 4)).

## Discussion

In this study, we showed the feasibility of a pilot 3-day course on antimicrobial stewardship aiming at primary-care physicians. Our data indicate the need for more AMS courses in this sector, along with the provision of primary-care specific guidelines/algorithms and the implementation of point-of-care diagnostics to facilitate the acceptance of AMS activities by primary-care physicians. Although primary-care physicians are increasingly being addressed in the context of AMS and AMR, as exemplified by the updated AMR national action plan of Germany [13], there is still a paucity of data on both AMS educational endeavors and interventions in the primary-care setting in Germany. 

Internationally, several AMS studies in the primary-care setting have been published. One stepped-wedge randomized-controlled trial from the US demonstrated that an education- and feedback-based intervention in primary care can reduce overall antibiotic prescribing in respiratory tract infections [14]. Another US study evaluated the effect of a multifaceted intervention aiming at respiratory tract infections and asymptomatic bacteriuria [15]. The intervention comprised individualized feedback including peer benchmarking, pocket cards with guidelines, clinical decision support sets, patient leaflets, and educational sessions. Although the endpoint of reduced overall antibiotic use was missed, a decrease in prescriptions for a subgroup of respiratory tract syndromes was noted. 

In contrast, a Swiss study failed to show an effect of regular audits with feedback and peer benchmarking among high-prescribing primary-care physicians [16].

McIsaac et al. from Canada conducted an interventional study including education on the most prevalent infectious diseases [17]. In addition, clinical decision support, patient leaflets, and financial incentives were implemented. Although there was no difference in overall antibiotic prescribing between control and intervention units, McIsaac et al. found a higher number of delayed prescriptions and a lower number of long antibiotic treatment durations (longer than seven days).

Another common strategy has been delayed prescribing for acute otitis media, and recent studies have demonstrated the feasibility of this low-cost intervention [18]. 

In contrast to the high demand for AMS in respiratory tract and urinary tract infections on an outpatient population level [7], our participants’ exam performances demonstrated an *a priori* higher expertise for urinary tract infections (Q3), compared with other topics.

The strengths of our study are the in-depth analysis including open text answers by the participants, allowing a qualitative assessment of what primary-care physicians need in order to perform AMS. Our data may help to address the barriers that were mentioned, e.g., the lack of expertise, tools, and time.

Our study has limitations that should be discussed. First, our course may have attracted primary-care physicians who already have a raised awareness for AMS, indicated by the proportion of the participants who had previously attended an AMS-related course. Reassuringly, the participants estimated “reduced AMR” and “optimized treatment” as similarly important. We anticipated and accepted this selection bias, in light of the voluntary nature of our pilot project. In addition, course fee was set at a low threshold (50 €) to enable a high participation rate. Second, the overall sample size and participation in the exams was low. Third, a testing effect may have biased the results of the post-course exam towards a seemingly better performance. Finally, our study lacks any measure of quality indicators pertaining to real-life antibiotic prescribing of the participating physicians. However, this was beyond the scope of this pilot educational project, but could be explored in a follow-up study.

We observed two recurring themes in the responses of our participants: the need for primary-care–specific guidelines and the perceived importance of diagnostics, especially as point-of-care. Therefore, guidelines that primarily aim at the primary-care sector should be developed, as demonstrated in some model projects in Germany [19]. The role of diagnostics, both with regard to pathogen testing and biomarkers, has recently been brought into focus [20]. A recent update of a Cochrane systematic review demonstrated the beneficial effect of C-reactive protein in the primary-care setting to guide the use of antibiotics [21]. However, there is little data on the use of procalcitonin, which has been implemented in secondary and tertiary care, as well as newer markers [22]. Hence, future studies should be specifically designed to include outpatients and those seen in primary care, where the majority of antibiotic use, including misuse, takes place.

Since we conducted our study, several projects have been initiated that employ novel digital tools, such as smartphone applications, to facilitate AMS interventions [23]. Artificial intelligence (AI)-based tools and their introduction into healthcare will likely propel AMS projects, also in the primary-care sector.

In order to achieve sustainable results, AMS in primary care needs to be adopted on different levels, including: reimbursement strategies and incentives, such as delayed prescription or withholding of antibiotics, with or without the use of proper pathogen and host diagnostics; the participation in AMR and antimicrobial-use surveillance programs that are already in place in Germany [24]; and the implementation of continuous education and feedback on prescription behavior. In any of these, AI-based tools may be of use. The inclusion of patient education and communication strategies between primary-care physicians and patients are additional approaches that warrant further evaluation in future studies [25].

## Notes

### Competing interests

The authors declare that they have no competing interests.

### Funding

This research project did not receive any specific grant from funding agencies in the public, commercial, or non-profit sectors.

### Acknowledgements

We thank Silke Mahler, Silke Müller, Roger Vogelmann, Sophie Schneitler, and Harald Böttge for their support in organizing and conducting the course.

### Data availability statement

The data that support the findings of this study are available from the corresponding author upon reasonable request.

## Supplementary Material

Course leaflet, questionnaire (only available in German)

## Figures and Tables

**Table 1:**
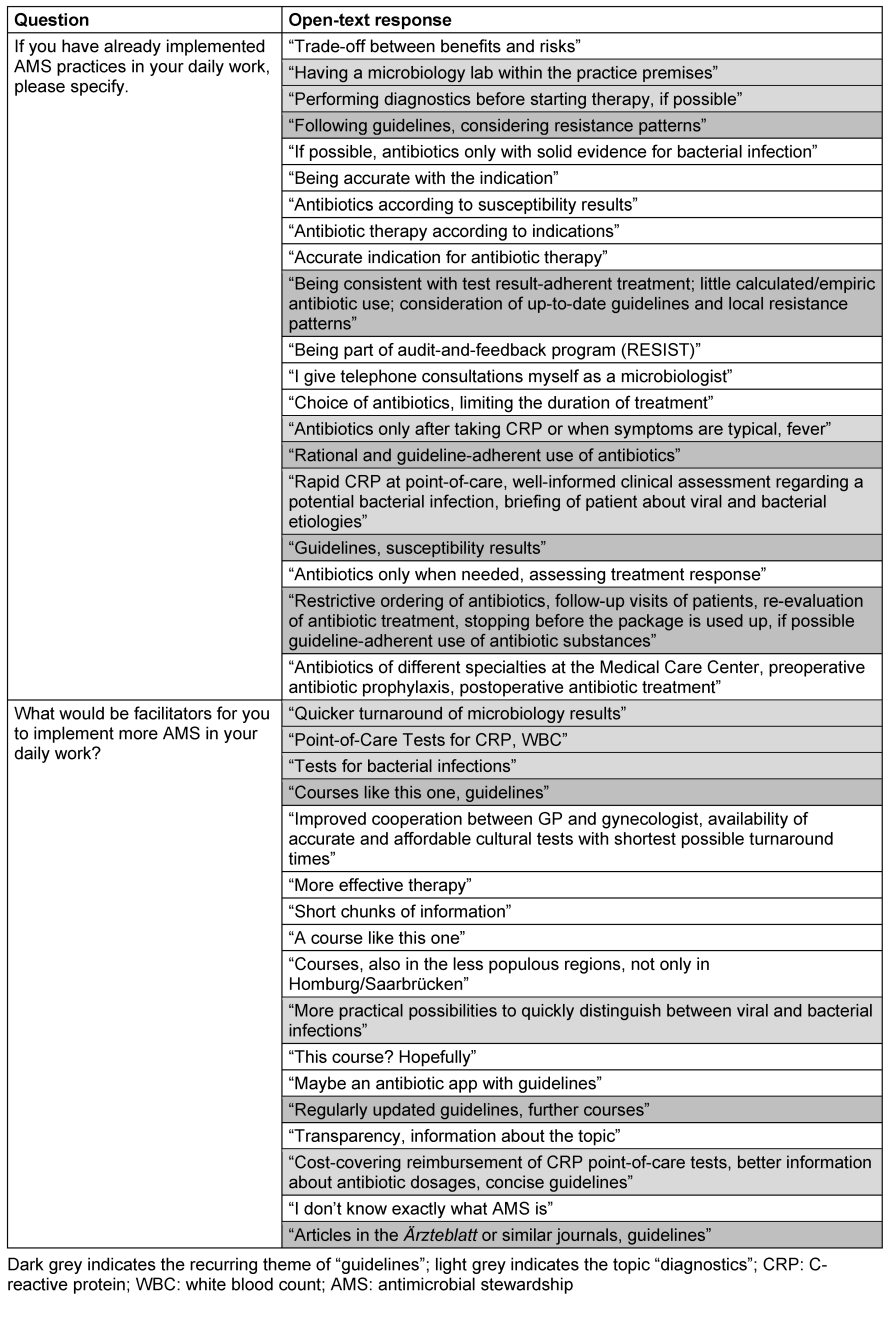
Open text responses to two questions regarding AMS implementation and facilitators
