# Supplementary material for: Attitudes of primary care physicians towards antimicrobial stewardship and the impact of a multi-part training course – a pilot study
Source: GMS Hyg Infect Control. 2023 Oct 10;18:Doc24. doi: 10.3205/dgkh000450 (PMC10665713; doi:10.3205/dgkh000450)
Supplement: Course leaflet, questionnaire (only available in German) [file HIC-18-24-s-001.pdf]

Referenten/Referentinnen

Prof. Dr. Dr. Sören L. Becker
Institut für Medizinische Mikrobiologie und Hygiene, Universitätsklinikum des Saarlandes

Dr. Harald Böttge
Gesundheitszentrum Ensheim

Katharina Last
Institut für Medizinische Mikrobiologie und Hygiene, Universitätsklinikum des Saarlandes

Dr. Sophie Schneitler
Institut für Medizinische Mikrobiologie und Hygiene, Universitätsklinikum des Saarlandes

Prof. Dr. Arne Simon
Kinderklinik,
Universitätsklinikum des Saarlandes

PD Dr. Roger Vogelmann
Mannheimer Onkologie Praxis
Universitätsmedizin Mannheim

Organisation

Katharina Last, Cihan Papan, Sören L. Becker
Universitätsklinikum des Saarlandes

& Ihr InfectioSaarNetz -Team

InfectioSaarNetz

Saarländisches Kompetenznetzwerk zur Infektionsprävention
Tel. 06841/16-23900, Mail: info@infectio-saar.de

www.infectio-saar.de

Die Teilnahmegebühr beträgt 50,00 €
für alle drei Termine.

Bitte senden Sie uns Ihre Anmeldung bis zum 12.01.2020 formlos per E-Mail (info@infectio-saar.de) oder Fax (06841-16 23985) unter Angabe folgender Informationen:

- Ihr Name
- Ihre Institution (Praxis, MVZ, …)
- Anzahl der Teilnehmer
- Ihre E-Mail-Adresse

Für Ärzte und Pharmazeuten wurden Fortbildungspunkte bei der Ärztekammer des Saarlandes beantragt.

Ministerium für Soziales, Gesundheit, Frauen und Familie SAARLAND

Bundesministerium für Gesundheit

InfectioSaarNetz

Einladung zur Seminarreihe

Antibiotic Stewardship (ABS) im ambulanten Bereich

Behandlungsprinzipien und -strategien für ambulante Infektionen

Mittwochs, 16:00 – 18:45 Uhr
22.01., 29.01. und 19.02.2020

Ärztekammer des Saarlandes
Faktoreistraße 4
66111 Saarbrücken

Sehr geehrte Damen und Herren,

wir laden Sie sehr herzlich zur

InfectioSaarNetz-Seminarreihe

am **22.01.**, **29.01.** und **19.02.2020** nach Saarbrücken ein.

Während dieser Veranstaltungsreihe möchten wir Sie über den rationalen und indikationsgerechten Einsatz von Antibiotika in der ambulanten Versorgung informieren und diese praxisnah mit Ihnen diskutieren.

In verschiedenen Vorträgen werden sowohl die theoretischen Grundlagen und Behandlungsprinzipien der ambulanten Antibiotikatherapie als auch die Anwendung bei konkreten, ambulanten Krankheitsbildern besprochen. Besondere Patientengruppen wie Kinder finden dabei ebenso Berücksichtigung. Abschließend gleichen wir das neu erworbene Wissen mit einer Abschlussdiskussion und einem TED-basierten Quiz ab.

Wir würden uns über Ihr Interesse und Ihre Teilnahme an dieser praxisnahen Veranstaltung mit hoher Relevanz für den klinischen Alltag sehr freuen. Auch eine Teilnahme an nur einzelnen Terminen ist möglich.

Mit herzlichen Grüßen

Prof. Dr. Dr. Sören Becker *Prof. Dr. Arne Simon*

Programmablauf I

InfectioSaarNetz - Seminarreihe

22.01.2020 **Teil 1**

16:00h – 16:15h Begrüßung und Überblick
Prof. Dr. Dr. Sören Becker &
Prof. Dr. Arne Simon

16:15h – 16:45h Antibiotic Stewardship: Konzept und Grundlagen
Katharina Last

16:45h – 17:15h Antibiotika in der Hausarztpraxis
PD Dr. Roger Vogelmann

Pause

17:35h – 18:05h Pharyngitis/Tonsillitis
PD Dr. Roger Vogelmann

18:05h – 18:45h Harnwegsinfektionen
PD Dr. Roger Vogelmann

29.01.2020 **Teil 2**

16:00h – 16:15h Begrüßung und Zusammenfassung der letzten Sitzung
Prof. Dr. Dr. Sören Becker

16:15 – 17:15h Atemwegsinfektionen: Bronchitis, Pneumonie und Rhinosinusitis
Dr. Sophie Schneitler

Programmablauf II

InfectioSaarNetz - Seminarreihe

(Fortsetzung)

Pause

17:35h – 18 :15h Sinnvolle Erregerdiagnostik und (lokale) Resistenzsituation
Prof. Dr. Dr. Sören Becker

18:15h – 18:45h Tools/Verschreibungsstrategien für die Praxis
Dr. Sophie Schneitler

19.02.2020 **Teil 3**

16:00h – 16:15h Begrüßung und Zusammenfassung der letzten Sitzung
Prof. Dr. Dr. Sören Becker

16:15h – 16:45h Haut-/Weichteilinfektionen
Dr. Harald Böttge

16:45h – 17:30h Antibiotikaeinsatz bei Kindern
Prof. Dr. Arne Simon

Pause

17:50h – 18:45h Abschlussdiskussion mit Quiz
Prof. Dr. Dr. Sören Becker

Attachment to: Last K, Simon A, Gärtner BC, Becker SL, Papan C. Attitudes of primary care physicians towards antimicrobial stewardship and the impact of a multi-part training course – a pilot study. GMS Hyg Infect Control. 2023;18:Doc23. DOI: 10.3205/dgkh000449

Fragebogen „Antibiotic Stewardship (ABS) im niedergelassenen Bereich“

Angaben zur Person

1. Geschlecht
    a. Weiblich ☐
    b. Männlich ☐
    c. Keine Angabe ☐

2. Altersgruppe
    a. 30 Jahre oder jünger ☐
    b. 31 – 40 Jahre ☐
    c. 41 – 50 Jahre ☐
    d. 51 – 60 Jahre ☐
    e. > 60 Jahre ☐

3. Berufsgruppe (bezogen auf Ihre primär ausgeübte Tätigkeit)
    a. Allgemeinmediziner/in, Hausarzt/Hausärztin ☐
    b. Hausärztlich tätige Internist/in ☐
    c. Internist/in ☐
    d. Dermatolog/in ☐
    e. HNO-Arzt/Ärztin ☐
    f. Pädiater/in ☐
    g. Chirurg/in ☐
    h. Gynäkolog/in ☐
    i. Apotheker/in ☐
    j. Andere: ___________________ ☐

Allgemeine Fragen

4. Wie/wo haben Sie von diesem Kurs erfahren? (Mehrfachangaben möglich)
    a. Ärzteblatt (gedruckt) ☐
    b. Internet ☐
    c. Email-Verteiler ☐
    d. Andere: _________________________________ ☐

5. Was bedeutet Antibiotic Stewardship (ABS) für Sie? (Mehrfachangaben möglich)
    a. Therapieoptimierung ☐
    b. Weniger Kosten ☐
    c. Kürzere Antibiotika-Therapie ☐
    d. Ich kann mir unter ABS bislang nichts Konkretes vorstellen ☐
    e. Sonstiges: _______________________________ ☐

6. Haben Sie in der Vergangenheit schon mal einen ähnlichen Kurs zum Thema ABS besucht?
    a. Ja ☐
    b. Nein ☐
    c. Sonstiges: _______________________________ ☐

7. Setzen Sie ABS in Ihrem täglichen ärztlichen Tun ein?
    a. Ja ☐
    b. Nein ☐
    c. Ich weiß nicht ☐

8. Wenn ja, inwiefern?

    a. ____

9. Wenn nein, was sind Ihrer Meinung hierfür die Gründe? (Mehrfachangaben möglich)
    a. Zu wenig Zeit ☐
    b. Keine Refinanzierung des Mehraufwandes ☐
    c. Zu wenig technische Ausstattung (z.B. Point of Care Testgeräte) ☐
    d. Zu wenig Kenntnisse ☐
    e. Patientenwunsch nach Antibiotika ☐
    f. Meine Patienten sind auch ohne ABS angemessen versorgt ☐
    g. Sonstiges: ____ ☐

10. Was würde Ihnen helfen, mehr ABS-bezogene Maßnahmen in Ihrem beruflichen Alltag zu implementieren?

    a. ____

11. Was ist/wäre die Hauptmotivation für Sie, ABS stärker in Ihr ärztliches Tun einfließen zu lassen? (Mehrfachangaben möglich)
    a. Bessere Therapie für meine Patienten ☐
    b. Kosten sparen ☐
    c. Vorteile für die Gesamtbevölkerung durch geringeren Selektionsdruck/geringere Resistenzen ☐
    d. Sonstiges: ____ ☐

12. Würden Sie folgende Angebote annehmen, um Ihre ABS-bezogenen Maßnahmen fortlaufend zu evaluieren und zu verbessern? (Mehrfachangaben möglich)
    a. Regelmäßige Rückmeldung meines Antibiotika-Rezeptierverhaltens im Vergleich mit anderen Praxen meines Fachgebietes ☐
    b. Nutzung einer Telefon-Hotline zur Besprechung von ABS-, infektiologischen und mikrobiologischen Problemen ☐
    c. Bereitstellung von aktuellen, kurz gefassten Handlungsalgorithmen und Leitlinien ☐
    d. Regelmäßige (z.B. jährliche) Fortbildungsveranstaltungen zum Thema ABS / Infektiologie ☐
    e. Sonstiges: ____ ☐

Anwendungsfragen

13. Es stellt sich ein 55jähriger sonst gesunder Mann vor mit Fieber, Husten und rechtsthorakalen, atemabhängigen Schmerzen. Die Auskultation ist unauffällig. Sie sind glückliche/r Besitzer/in eines Point-of-care-Gerätes und veranlassen eine Bestimmung des C-reaktiven Proteins, welches 100 mg/L beträgt. Im Röntgen Thorax, welches in der im selben Gebäude ansässigen radiologischen Praxis durchgeführt wird, wird ein beginnendes Mittellappeninfiltrat beschreiben. Sie diagnostizieren eine ambulant-erworbene Pneumonie und entscheiden sich aufgrund des guten Allgemeinzustandes zunächst zur ambulanten Therapie. Welches Antibiotikum würden Sie verschreiben?
    a. Azithromycin ☐
    b. Ciprofloxacin ☐
    c. Amoxicillin ☐
    d. Cefuroxim ☐
    e. Fosfomycin ☐

14. Eine 35jährige sonst gesunde Frau stellt sich mit Husten, Schnupfen und Halsschmerzen vor. In ihrer Familie (Zwillinge im Alter von 2, Ehemann) hätten alle ähnliche Symptome, die Kinder hätten zusätzlich gerötete Augen. Bei der Inspektion fällt Ihnen eine Rötung des Rachens sowie vereinzelte weißliche Stippchen auf den Mandeln auf. Wie gehen Sie vor?
    a. Abstrich auf Streptokokken abnehmen, Ergebnis abwarten ☐
    b. Abstrich auf Streptokokken abnehmen, Penicillin beginnen ☐
    c. Serologie auf Epstein-Barr-Virus, keine Antibiotika ☐
    d. Überprüfen des Diphtherie-Impfschutzes, Penicillin beginnen ☐
    e. Symptomatische Therapie einleiten, keine Antibiotika ☐

15. Eine 24jährige sonst gesunde Frau stellt sich nach ihren Flitterwochen mit Brennen und häufigem Wasserlassen in Ihrer Praxis vor. Die Patientin hat kein Fieber oder vaginalen Ausfluss. Sie habe versucht, sich mit reichlich Cranberry-Saft zu behandeln, was aber nicht geholfen habe. Welches Antibiotikum würden Sie am ehesten verschreiben?
    a. Fosfomycin ☐
    b. Cefuroxim ☐
    c. Ciprofloxacin ☐
    d. Cefpodoxim ☐
    e. Amoxicillin ☐

16. Es wird Ihnen ein 5jähriger Junge mit folgendem Hautbefund vorgestellt (Abbildung 1).

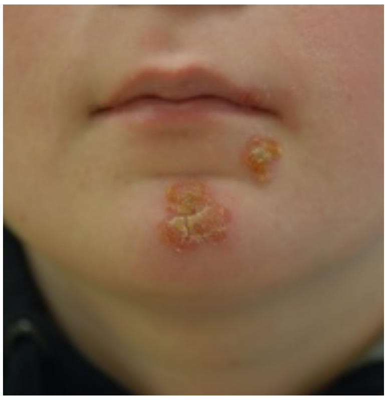

Abbildung 1. © Dietrich Abeck; https://www.pharmazeutische-zeitung.de/ausgabe-382011/bakterien-zoo-ausser-kontrolle/

Das übrige Integument ist unauffällig. Fieber wird verneint. Der Junge hat keine Vorerkrankungen und sonst auch keine Beschwerden. Welche Therapie empfehlen Sie?

   a. Lokale Therapie mit Mupirocin ☐
   b. Lokale Therapie mit Octenisept ☐
   c. Cefuroxim p.o. ☐
   d. Sultamicillin / Ampicillin-Sulbactam p.o. ☐
   e. Lokale Therapie mit Fusidinsäure ☐

17. Ein 68-jähriger Mann stellt sich in Ihrer Praxis vor und gibt wechselhafte Beschwerden bei der Miktion sowie gelegentliche Schmerzen im Perinealbereich an. Er habe von seinem Vorbehandler vor einem Jahr eine "Antibiotika-Kur" mit Levofloxacin über 6 Wochen erhalten, wodurch eine leichte, vorübergehende Verbesserung aufgetreten sei. Er bittet Sie nun um eine Wiederholung der Antibiotika-Therapie, diagnostische Maßnahmen wünscht er nicht. Halten Sie die Indikation für eine antibiotische Therapie für gerechtfertigt?

   a. Ja ☐
   b. Nein ☐
   c. Ich weiß nicht/unsicher ☐

18. Wie wahrscheinlich ist es, dass Sie in dieser Situation ein Antibiotikum verschreiben würden? (Auf einer Skala von 1 bis 5, 1= extrem unwahrscheinlich, 5= extrem wahrscheinlich)

   a. 1 ☐
   b. 2 ☐
   c. 3 ☐
   d. 4 ☐
   e. 5 ☐
